# Growth Mindset and Job Crafting: A Trait Activation Perspective with Job Autonomy as Moderator

**DOI:** 10.3390/bs14121221

**Published:** 2024-12-18

**Authors:** Tao Yu, Lidong He, Hu Ying, Jie Liu, Yuzhen Wu, Yun Wang, Xiaofu Pan

**Affiliations:** College of State Governance, Southwest University, Chongqing 400715, China; lhr@swu.edu.cn (T.Y.);

**Keywords:** growth mindset, job crafting, job autonomy, trait activation theory, positive organizational behavior

## Abstract

Job crafting benefits both employees and organizations by enhancing employees’ health, well-being, and performance. Therefore, it is crucial to investigate the individual factors that encourage job crafting and the conditions under which they operate. Based on Trait Activation Theory, this study examined the relationship between employees’ growth mindset and job crafting, as well as the moderating effect of job autonomy on this relationship. In Study 1, we conducted a situational experiment with 180 participants, manipulating growth vs. fixed mindset and high vs. low job autonomy. In Study 2, we surveyed 236 participants over three waves, collecting demographic data and growth mindset at T1, job autonomy at T2 (one month later), and job crafting at T3 (two months later). Results from Study 1 indicated that growth mindset significantly impacted job crafting, moderated by job autonomy. Study 2 confirmed this positive effect of growth mindset on job crafting and revealed a significant positive interaction between growth mindset and job autonomy. These findings suggest that employees with a growth mindset engage more in job crafting, with this relationship strengthened under conditions of high job autonomy. This study highlights job autonomy as a situational cue that activates employees’ growth mindset, enhancing proactive job crafting behaviors. This research advances the literature on positive work behaviors by establishing growth mindset as a direct antecedent and illustrating the moderating role of job autonomy, thereby enriching the understanding of conditions that foster a positive workplace environment.

## 1. Introduction

With the rapid advancement of technologies such as artificial intelligence, automation, and digital platforms, traditional top-down job designs are increasingly insufficient to meet the dynamic demands of modern workplaces [1]. These technologies not only reshape the nature of tasks but also necessitate greater flexibility and adaptability in job roles. In response, employees are expected to proactively redesign their work to align with evolving organizational goals and technological requirements [2]. This shift highlights the growing importance of job crafting as a bottom-up strategy for job redesign [3,4], where individual traits such as a growth mindset and contextual factors like job autonomy play critical roles.

Job crafting holds significant implications for both individuals and organizations. For individuals, it could enhance health [5], well-being [6,7], and career success [8,9], while reducing job burnout [10] and negative emotions [8,11]. For organizations, employees who engaged in job crafting exhibited greater work engagement [10,12,13,14], job satisfaction [15], organizational citizenship behavior [16,17], innovation [18], and job performance [13,14,19]. Additionally, they demonstrated lower turnover intentions [20,21] and counterproductive behaviors [22]. Consequently, promoting employees’ job crafting has become a pivotal issue in organizational research and practice.

As job crafting is a positive organizational behavior at the individual level [4], researchers have been particularly interested in which personal factors influence job crafting. Some researchers focused on the stable individual traits such as employees’ Big Five personality traits [23], proactive personality [19], and regulatory focus [24]. Others concentrated on individual motivational factors such as self-efficacy [25] and core self-evaluation [26]. In recent years, researchers began to study the relationship between growth mindset and job crafting. Growth mindset is an individual’s belief that self-attributes, such as intelligence, ability, morality, and personality, can be changed through effort [27]. Previous research showed a positive correlation between growth mindset and job crafting, including cognitive, skill, and overall job crafting [28].

Yet, there remain noteworthy issues to be explored. For example, previous studies treated employees’ growth mindset as a moderating variable, ignoring its direct influence on job crafting [28]. Additionally, while a study revealed that employees’ growth mindset of job characteristics (job characteristic can be changed) positively impacted their willingness to craft their jobs [6], they did not explore the direct impact of growth mindset of personal attributes (e.g., work ability can be changed) on job crafting. Job crafting can reflect various changes in work ability, including both long-term and short-term aspects. Long-term changes, such as those related to aging, skill erosion, or gradual changes in work ability, require employees to adapt their roles and tasks to align with evolving capabilities. On the other hand, short-term changes—such as those caused by unexpected challenges, new job demands, or immediate shifts in work context—necessitate quick adaptations and proactive behaviors.

Moreover, previous research neglected to investigate the conditions under which employees’ growth mindset impacted their job crafting. Job crafting, although a proactive individual-level behavior, was also influenced by organizational context and contextual factors [29]. Therefore, scholars advocated exploring the interactive effects of contextual and individual factors on job crafting [29,30]. For instance, job autonomy, as a contextual factor, moderated the relationship between proactive personality and job crafting. Specifically, this relationship was stronger when employees perceived higher job autonomy [31]. In view of this, it is plausible that contextual factors may also moderate the relationship between employees’ growth mindset and job crafting.

To address these questions, this study aimed to explore whether and under what conditions employees’ growth mindset impacted job crafting based on Trait Activation Theory (TAT) [32,33]. TAT provides a comprehensive framework for understanding the interaction between individual traits and contextual factors in influencing behavior [32]. By identifying situational cues that activate specific traits, TAT offers a nuanced lens through which to examine proactive work behaviors like job crafting. This theory aligns with our research objectives by addressing how growth mindset, a stable individual trait, is expressed under varying levels of job autonomy, a situational cue.

This study comprises two distinct but complementary approaches: Study 1, a situational experiment, was conducted to establish causal relationships between growth mindset, job crafting, and job autonomy under controlled conditions. Study 2, a longitudinal survey, aimed to replicate these findings in a real-world setting and enhance ecological validity. The combination of these methods strengthens the robustness of the conclusions and provides a comprehensive understanding of the phenomena under investigation.

## 2. Literature Background and Hypothesis Development

### 2.1. Job Crafting

Researchers have variously defined job crafting from different theoretical perspectives. Wrzesniewski and Dutton first proposed this construct from the perspective of job roles, defined job crafting as follows: “employees gain job identity and job roles by crafting their own jobs” [4]. In other words, employees could spontaneously and actively adjust their work tasks according to their own preferences, personalities, abilities, etc. They believed that job crafting included three dimensions: task crafting, relational crafting, and cognitive crafting.

Tims and Bakker, then, defined job crafting as an employee’s behavioral change, based on his/her own abilities and demand preferences [3], aimed at balancing job demands and resources based on the perspective of a job demands–resource model [34]. They identified three types of job crafting: increasing job resources, increasing job demands, and decreasing job demands. Tims et al. further expanded this categorization into four types: increasing social job resources, increasing structural job resources, increasing challenging job demands, and reducing hindering job demands [12].

In recent years, researchers adopted approach–avoidance motivation theory or promotion–prevention regulatory focus theory as a novel, integrated framework for understanding job crafting. For example, Bruning and Campion categorized job crafting into two types, approach crafting and avoidance crafting, further dividing each into two components: role crafting and resource crafting [35]. Zhang and Parker similarly classified job crafting into approaching crafting and avoidant crafting, under which two types of job crafting include behavioral crafting and cognitive crafting, respectively [30]. Bindl et al. classified job crafting into promotion-oriented crafting and prevention-oriented crafting, with each category including task crafting, relationship crafting, skill crafting, and cognitive crafting [36]. In other words, promotion-oriented job crafting emphasizes increasing positive job characteristics, demands, and outcomes, while prevention-oriented crafting job crafting focuses on avoiding negative ones. This study, therefore, focuses on promotion-oriented job crafting.

Furthermore, the adoption of new technologies accelerates the transformation of work environments, creating opportunities for innovative behaviors while also introducing challenges such as skill obsolescence and job displacement [1]. Understanding how employees’ growth mindset interacts with contextual cues like job autonomy to foster proactive behaviors is therefore crucial for designing resilient and adaptive workplaces in the face of technological disruption.

### 2.2. Employees’ Growth Mindset and Job Crafting

Mindset is defined as a continuum of meaning systems that organized concepts such as goals, attributions, helplessness, and effort beliefs, with a fixed mindset at one end of the continuum and a growth mindset at the other [37]. A growth mindset is the belief that personal attributes, such as intelligence, ability, morality, or personality, can be changed through effort; conversely, a fixed mindset is the belief that these attributes are innate or unchangeable [27,37]. Individuals with a growth mindset exhibited higher learning motivation, positive emotion, and academic achievement [38,39,40,41]. They also showed greater prosocial motivation and behaviors in interpersonal and social interactions [42,43,44], as well as higher levels of health and well-being [45,46].

In the field of organizational psychology, researchers have found positive associations between employees’ growth mindset and work-related aspects, including motivation, behavior, and performance. For example, employees’ growth mindset predicted increased work engagement [47] and decreased job burnout [48]. Moreover, employees’ growth mindset positively predicted their positive organizational behaviors. In terms of in-role behaviors, growth-mindset employees experienced less goal disengagement [49], resulting in more error-learning behaviors [50] when receiving negative feedback. In terms of extra-role behaviors, growth-mindset employees had more helping, voice, and organizational citizenship behaviors [51]. Furthermore, employees’ growth mindset positively impacted job performance. Salespeople who believed in the malleability of sales ability demonstrated greater sales confidence, were less likely to avoid performance goals or performance feedback [52], and consequently achieved higher sales performance [53].

Given that growth-mindset individuals had more positive cognitive, affective, and behavioral responses to challenges [27], this study suggests a positive association between growth mindset and job crafting. On the one hand, growth-mindset employees believe that their work ability can be improved through effort. This belief is crucial for job crafting, as it involves employees redesigning their jobs based on their work ability [4]. Those who believe in the malleability of their work ability hold stronger effort beliefs [54]. Even if their current abilities do not fully meet job demands, they are confident they can develop them through efforts like enhancing job resources and requirements. Conversely, if employees perceive their work ability as fixed, they are more likely to stick to tasks within their current abilities. Attempting to expand tasks or build new relationships could expose their limitations, potentially damaging their self-esteem [55].

On the other hand, growth-mindset individuals are more willing to seek challenges, which is a distinctive feature of job crafting. They tend to perceive difficulties and challenges as opportunities for learning and growth [27], making them more likely to pursue demanding tasks [40]. Researches showed that growth-mindset employees sought more work-related challenges, such as innovation at work by utilizing their strengths or feedback from others [56], and learned from errors [50,57]. Similar to innovation and error learning, job crafting involves risks and challenges, such as the possibility of being stretched beyond one’s current work ability or failing to meet job goals while increasing challenging job demands. There is also the risk of communication failures in relational crafting, leading to rejection by others. Therefore, growth-mindset employees are more likely to embrace job crafting as an opportunity for learning and personal development. These arguments suggest that employees with a growth mindset are more likely to engage in proactive work behaviors such as job crafting. Therefore, we propose the following:

**Hypothesis 1.**  *Employees’ growth mindset positively relates with job crafting.*

### 2.3. The Moderating Role of Job Autonomy

According to TAT, traits latent within an individual are expressed as specific explicit behaviors only when these traits are aroused or activated in certain contexts [32]. The contextual factors that trigger trait expression are known as trait-relevant situational cues, and the resulting behaviors are termed trait-expressive behaviors. Trait-relevant situational cues influence personality expression in five ways: (1) demands, which are cues that afford opportunities for individual to engage in positively valued ways; (2) distracters, which are cues that negatively affect individuals’ work behavior or performance; (3) constraints, which are cues that restrict the expression of traits, thereby inhibiting their influence on behavior or performance; (4) releasers, which are cues related to discrete work events that can counteract the effects of constraints; (5) facilitators, which are cues that may enhance behavioral expression by highlighting trait-relevant information already present in a certain context [33]. Given this, job autonomy, as a trait-relevant situational cue, can function as a “demand” in the trait activation process of growth mindset.

In addition to individual characteristics and motivational factors, job crafting is also influenced by contextual factors. Lin and Meng clearly stated that job crafting was essentially a contextual proactive behavior [29]. On the one hand, social context is both a target and a component of job crafting; on the other hand, social contextual factors directly influence job crafting and interact with other individual characteristics to influence job crafting. Therefore, this study also focused on the interaction of social contextual factors and employees’ growth mindset on job crafting.

Therefore, we argue that job autonomy enhances the positive relationship between employees’ growth mindset and job crafting. Job autonomy is the extent to which employees make autonomous choices and decisions about work methods, work scheduling, and work criteria [58]. Job autonomy may encourage and support employees to translate the belief that work ability can be changed through effort into specific work behaviors. Job autonomy is a well-established contextual factor influencing various workplace behaviors. Prior studies have demonstrated that job autonomy moderates the relationship between individual traits, such as proactive personality, and job crafting, strengthening this relationship when autonomy levels are high [31]. Similarly, in the context of a growth mindset, high job autonomy provides opportunities for employees to apply their belief in the malleability of abilities by actively engaging in proactive behaviors [59,60]. By empowering individuals to pursue challenges and customize their roles, job autonomy serves as a situational cue that activates growth mindset and facilitates job crafting. These findings underscore the importance of job autonomy as a critical moderator in the growth mindset–job crafting relationship.

Employees with higher job autonomy are better able to utilize a growth mindset, as their independence and freedom in the workplace empower them to develop skills and seek challenges independently [30]. Specifically, higher job autonomy reduced the constraints of work methods, procedures, and effort, and empowered employees with greater freedom and more opportunities to increase tasks, build relationships, and rethink the meaning of work [61]. Thus, job autonomy strengthens the positive associations between employees’ growth mindset and job crafting. Conversely, lower levels of job autonomy require employees to adhere strictly to prescribed work methods, scheduling, and criteria [58], which may hinder learning and networking opportunities [30]. Even if they believe their work ability can improve, they may lack opportunities to refine work methods, streamline procedures, or pursue challenging tasks.

In a word, job autonomy, as a situational cue, activates employees’ growth mindset, facilitating proactive behaviors like job crafting. High levels of autonomy provide employees with the freedom to apply and develop their skills, strengthening the relationship between growth mindset and job crafting. Thus, we propose the following:

**Hypothesis 2.**  *Job autonomy moderates the positive relationship between employees’ growth mindset and job crafting, with the strength of this relationship increasing with higher autonomy and decreasing with lower autonomy.*

## 3. Study 1: Situational Experiment

### 3.1. Methods

#### 3.1.1. Participants

We recruited full-time employees as participants through Credamo (Creator of Data and Model), a one-stop smart research platform. The informed consent form outlined the research introduction, process, potential risks or discomforts, compensation, and benefits. Two hundred employees participated in this study and confirmed that they “have read and understood the informed consent form and voluntarily agreed to participate in this study.” We removed study data that were not answered carefully through attention testing questions and ended up with 180 samples. Of these, 32.2% were male and 67.8% were female, with an average age of 31 years (SD = 8.85). Participants had varying educational backgrounds, with 73% holding a bachelor’s degree or higher.

#### 3.1.2. Experimental Materials

Manipulation materials for employees’ mindset. Drawing from the classic experimental manipulation paradigm of growth mindset established by Dweck [62] and following the methods of prior researchers like Chiu et al. [63], we employed reading articles to manipulate the mindsets of our participants. Specifically, we created articles designed to manipulate the growth mindset and fixed mindset of employees by adapting manipulation materials originally used for mindsets of empathy [64]. For instance, the growth-mindset article referenced research by experts who maintain that work abilities are malleable, stating that “Work abilities are changeable and can be improved over time. They are not stable throughout a person’s career and can be enhanced and nurtured”. Conversely, the fixed-mindset article quoted experts who argued that work abilities were fixed, stating that “Work abilities are fixed and follow a consistent trajectory over time. While they may appear flexible initially, they eventually solidify to a point where they cannot be changed”.

The material introducing high job autonomy presented a work situation with significant autonomy: “Due to work requirements, you have been reassigned to a new position. In this job, you are free to determine your working time, scheduling, and method. Although the company operates from 8:00 a.m. to 5:00 p.m., you can arrange your commuting, meal, and post-work rest times based on your work demands. Additionally, depending on work demands, you have the flexibility to work remotely or in other locations. You are not required to report every work decision to your supervisor and can operate independently, making decisions in the ways and processes you are comfortable with, provided your methods adhere to legal and ethical standards”.

The material introducing low job autonomy presented a work situation with limited autonomy: “Due to work requirements, you have been reassigned to a new position. In this job, you are required to adhere strictly to set working time, scheduling, and method. You must clock in at 8:00 a.m. daily, take lunch and breaks from 12:00 p.m. to 2:00 p.m., and clock out at 6:00 p.m. During working time, you must remain in the designated workplace and are not permitted to leave without permission. Additionally, every work decision you make must be approved by your supervisor before implementation. Your work execution must comply with the work code outlined in the company’s employee handbook, and prescribed work procedures cannot be arbitrarily altered”.

#### 3.1.3. Measures

Growth mindset: We employed the 8-item Chinese version Employee Growth Mindset Scale to measure employees’ growth mindset of work ability. Four items represented growth mindset (e.g., “No matter who I am, I can significantly change my basic level of work ability”), while the remaining four items represented a fixed mindset (e.g., “My work ability is fixed and I cannot change it”). This scale adopted a 6-point Likert format, ranging from 1 (strongly disagree) to 6 (strongly agree). Higher aggregated scores, with fixed-mindset items reversed, indicated a stronger growth mindset of work ability. Yu et al. reported a Cronbach’s α of 0.92 for the scale [65]. In this study, the Cronbach’s α was 0.96.

Job autonomy: We employed the 3-item Job Autonomy Subscale of the Psychological Empowerment Scale [61] to measure job autonomy. A sample item was “I can decide for myself how to proceed with my work.” This scale adopted a 5-point Likert format, ranging from 1 (strongly disagree) to 5 (strongly agree), with higher aggregated scores indicating a higher level of job autonomy. Li et al. reported a Cronbach’s α of 0.75 for the Chinese version [66]. In this study, the Cronbach’s α was 0.96.

Job crafting: Referring to the method employed by Liu and Wan [67], we employed the revised 4-item Job Crafting Scale to measures job crafting in the envisioned work situation [68]. A sample item was “In this job, I will take the initiative to introduce new methods to improve my work.” This scale adopted a 6-point Likert format, ranging from 1 (strongly disagree) to 6 (strongly agree), with higher aggregated scores indicating a higher degree of job crafting. Liu and Wan reported a Cronbach’s α of 0.91 for the Chinese version [67]. In this study, the Cronbach’s α was 0.92.

#### 3.1.4. Procedures

All participants were randomly assigned to 2 growth-mindset groups and 2 fixed-mindset groups. They read an informed consent form and a 1500-word article. The article was written by the authors but presented to the participants as a science article from *Science Daily* about the results of scientific research on the growth mindset (or fixed mindset). The article was divided into 5 parts, with a minimum dwell time of 30 s per page to ensure careful reading. After each part, participants responded to comprehension questions (serving as attention tests), such as “Classical organizational management research has convinced that: A. work abilities continue to change over time; B. work abilities remain stable over time; and C. whether work abilities change is environmentally related.” Incorrect answers to 2 or more questions indicated lack of careful reading. (In this question, growth-mindset groups should choose A, fixed-mindset groups should choose B, while C is a distraction.)

After reading the articles, participants completed the Employee Growth Mindset Scale. They then read the work autonomy material and imagined working in the new situation, describing it in at least 10 words. Next, they rated the imagined work situation using the Work Autonomy Scale. Finally, participants filled out the Job Crafting Scale and demographic information. To mitigate potential negative impacts, participants in fixed-mindset groups were told that “The materials used in this study were fictionalized! In fact, everyone’s work ability is changeable, and can be improved if they put in the effort, find the right strategies, and seek help from their leaders and coworkers”.

### 3.2. Results

#### 3.2.1. Manipulation Test

The validity of the experimental manipulation was tested by the Employee Growth Mindset Scale and the Job Autonomy Scale, as shown in Table 1. There were 180 participants, of which 83 participants were assigned to the growth-mindset groups, with a higher growth mindset rate (M = 5.07, SD = 0.64), while 97 participants were assigned to the fixed-mindset groups, with a lower growth mindset rate (M = 2.75, SD = 0.91), indicating that we successfully manipulated the growth and fixed mindset.

We then examined the mean score on the Job Autonomy Scale. As intended, the high job autonomy conditions resulted in a higher job autonomy rate (M = 4.48, SD = 0.37) than the low job autonomy conditions (M = 1.73, SD = 0.72), indicating that we successfully manipulated the high and low job autonomy.

#### 3.2.2. Main Effects and Interaction Effects

An analysis of variance (ANOVA) was conducted with the growth mindset as the independent variable; the job autonomy group as the moderating variable; gender, age, and education as covariates; and job crafting as the dependent variable. The results showed a significant main effect of growth mindset on job crafting, F (1, 174) = 17.24, *p <* 0.001, partial ƞ^2^ = 0.09, and the moderating effect of job autonomy was also significant, F (1, 174) = 18.25, *p <* 0.001, partial ƞ^2^ = 0.10.

Further simple effect analyses of the moderating effect showed that in the low job autonomy groups, the effect of growth mindset on employees’ job crafting was not significant (M _fixed mindset_ = 2.47, SD = 0.91; M _growth mindset_ = 2.47, SD = 1.22), F (1, 174) = 0, *p* = 0.989, partial ƞ^2^ = 0. While in the high job autonomy groups, the positive effect of growth mindset on employees’ job crafting was significant (M _fixed mindset_ = 4.15, SD = 0.73; M _growth mindset_ = 5.23, SD = 0.47), F (1, 174) = 37.85, *p <* 0.001, partial ƞ^2^ = 0.18. Detailed results are shown in Table 2.

## 4. Study 2: Three-Wave Survey

### 4.1. Methods

#### 4.1.1. Participants and Procedures

We recruited full-time employees as participants through Credamo. The informed consent form outlined this study’s purpose, process, risks, benefits, confidentiality, and withdrawal rights. Five hundred participants consented to participate after reading and understanding the form. Demographic variables and growth mindset were measured in T1. After excluding low-quality responses, such as those with a clear tendency to respond consistently, reverse scoring questions, and attention questions, 484 valid questionnaires were received, representing a 96.80% return rate. One month later (T2), we invited these 484 participants to complete job autonomy scale, resulting in 374 valid responses (77.27% recovery rate). One month after the second survey (T3), we invited the 374 participants from the second survey to respond to job crafting scale and received 236 valid responses (63.10% recovery rate). Overall, the three surveys had a combined effective recovery rate of 47.20%.

Participant demographics are summarized in Table 3. The final sample included 32.6% males and 67.4% females, with an average age of 31.87 years (SD = 6.35). Regarding educational background, 69.5% had a bachelor’s degree, and 21.6% held a master’s degree or higher.

#### 4.1.2. Measures

Employee Growth Mindset Scale (T1): Same as Study 1. The Cronbach’s α in this study was 0.92.

Job Autonomy Scale (T2): Same as Study 1. The Cronbach’s α in this study was 0.71.

Job Crafting Scale (T3): For this study, the portion of Bindl et al.’s job crafting scale focusing on promoting-oriented job crafting was utilized [36]. This portion of the scale consists of 4 parts: 4 items for task crafting (e.g., “I actively took on more tasks in my work”), 4 items for relationship crafting (e.g., “I actively sought to meet new people at work”), 4 items for skill crafting (e.g., “I actively tried to develop wider capabilities in my job”), and 4 items for cognitive crafting (e.g., “I tried to think of my job as a whole, rather than as separate tasks”). Participants rated their behaviors on a 5-point Likert scale (1 = not at all, 5 = a great deal), reflecting their frequency of job crafting in the past week. Higher scores indicate greater levels of promoting-oriented job crafting. This scale had good reliability [36]. In this study, the Cronbach’s α for the promoting-oriented job crafting scale was 0.87.

### 4.2. Results

#### 4.2.1. Descriptive Statistics and Correlation Analysis

Descriptive statistics of the main variables of this study were conducted. Means, standard deviations, and correlation coefficients of the variables with each other are shown in Table 4. Correlation analysis revealed that growth mindset was significantly positively correlated with job autonomy (r = 0.39, *p <* 0.01) and job crafting (r = 0.42, *p <* 0.01); job autonomy was also significantly positively correlated with job crafting (r = 0.51, *p <* 0.01). These results align with our theoretical expectations and provide initial support for our research hypotheses, paving the way for further hypothesis testing.

#### 4.2.2. Hypothesis Testing

The research hypotheses were tested using Model 1 of SPSS Macro PROCESS 2.13 [69]. Results are shown in Table 5. Regression analysis revealed that control variables, such as gender, age, and education, did not significantly impact job crafting, as shown in Table 5 (Model 1). However, controlling for these variables, employees’ growth mindset emerged as a significant positive predictor of job crafting (β = 0.42, SE = 0.04, *p <* 0.001), as shown in Table 5 (Model 2), indicating that the higher the level of employees’ growth mindset, the higher the level of job crafting.

To further test the moderating effect of job autonomy, an interaction between growth mindset and job autonomy was constructed. By incorporating this interaction into Model 2, Model 3 was derived. In Model 3, the interaction exhibited a statistically significant positive impact on job crafting (β = 0.17, SE = 0.05, *p <* 0.001), after considering the control variables.

**Figure 1:**
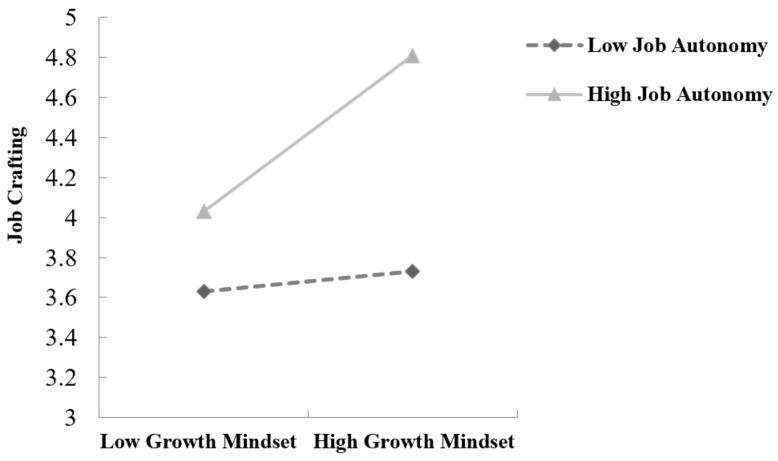
Simple slope analysis of job autonomy moderating the relationship between growth mindset and job crafting.

To explore the varying effects of growth mindset on job crafting across different job autonomy levels, a simple slope analysis was carried out, visualized in Figure 1.

As shown in Figure 1, the interaction effect between growth mindset and job autonomy on job crafting was significant. Specifically, the figure illustrates how the relationship between growth mindset and job crafting becomes stronger as job autonomy increases. At low levels of job autonomy, the effect of growth mindset on job crafting is minimal (β = 0.05, *p <* 0.001). However, at high levels of job autonomy, the positive impact of growth mindset on job crafting is considerably more pronounced (β = 0.39, *p <* 0.001). This suggests that job autonomy acts as a key moderator in the relationship between growth mindset and job crafting.

## 5. Discussion

Through two studies, a situational experiment and a questionnaire, we investigated the direct influence of employees’ growth mindset on job crafting and the moderating effect of job autonomy. The experimental results revealed that individuals with manipulated growth mindset were more inclined to craft their jobs in an imagined work situation compared to those with a fixed mindset. Additionally, the questionnaire data corroborated our hypothesis, indicating a direct positive impact of growth mindset on job crafting, enhancing the ecological validity of the results. Consistent with prior research [28], our findings underscored the positive association between growth mindset and job crafting. Both experimental and correlational evidence suggested that employees with a growth mindset exhibited higher levels of job crafting, encompassing task, relationship, skill, and cognitive crafting.

This study further investigated how job autonomy influenced the relationship between employees’ growth mindset and job crafting. In the experimental study, we found that growth mindset alone did not significantly impact job crafting in low-autonomy conditions, but it did in high-autonomy conditions. In the questionnaire study, we observed a positive moderation effect of job autonomy on the relationship between growth mindset and job crafting. Specifically, growth mindset positively influenced job crafting across all autonomy levels, but the effect was stronger in high-autonomy environments. Both studies suggest that job autonomy positively enhances the positive relationship between employees’ growth mindset and job crafting.

### 5.1. Theoretical Significance

First, this study enhances understanding of job crafting by exploring the role of employees’ growth mindset. Unlike previous studies that emphasized Big Five personality traits [23], proactive personality [31,70], regulatory focus [24,71,72], and self-efficacy [25,73,74], this study focuses on the direct impact of growth mindset on job crafting. While a growth mindset has often been treated as a moderating variable [28], this study establishes its direct significance in shaping job crafting. The experiment and questionnaire revealed that employees’ growth mindset positively influenced job crafting, bridging the gap in research on its direct effects and enriching knowledge on the factors that shape job crafting.

Second, this study further enriches the literature on growth mindset theory. Currently, growth mindset research is predominantly focused in educational and social psychology, with limited exploration in organizational psychology. By investigating the impact and mechanism of employees’ growth mindset on job crafting, this study extends growth mindset research into the field of organizational psychology. Additionally, we introduced novel research methods in this field, combining both situational experiment and questionnaire. This approach addresses concerns raised by researchers regarding the methodology used in growth mindset research in organizational settings [75,76].

Third, this study enhances the literature on TAT by validating the moderating role of job autonomy. Prior research in education has established that growth mindset’s positive impact on student motivation and behavior is moderated by psychological affordance [77], a situational characteristic. For instance, students with a growth mindset tend to excel in threatening or stereotype-threatening situations [78] and when social contexts are supportive [40]. Drawing from TAT [33], our study similarly demonstrates that the influence of employees’ growth mindset on work behavior depends on the activation of relevant contextual cues. Notably, job autonomy acts as a trigger for trait expression, adding depth to research on contextual cues within TAT. Specifically, job autonomy positively moderated the effects of employees’ growth mindset on job crafting. When job autonomy was limited, it became challenging for employees to activate their growth mindset and engage in trait-expressive behaviors. Conversely, a high degree of job autonomy prompted the activation of growth mindset, leading to more trait-expressive behaviors. Therefore, job autonomy serves as a “demanding” role for the growth mindset, positively enhancing its expression among employees and amplifying its influence on job crafting. This finding further enriches the literature on TAT.

### 5.2. Practical Significance

On the one hand, organizations should recruit and select employees with a growth mindset, as these employees are more likely to engage in job crafting and seek challenges. Given the increasing instability and complexity in the workplace, encouraging employees to voluntarily reshape their roles is essential. However, a mourning culture has led to a rise in employees adopting a passive “lying flat” attitude, particularly those with fixed mindsets [79]. This study found that employees with a growth mindset were more likely to seek challenges, take on additional tasks, improve communication, develop skills, and discover work meaning. Hence, organizations should select and cultivate growth-mindset employees for innovative roles. Training programs aimed at fostering growth mindsets could include modules on adaptive learning, resilience, and proactive behaviors.

On the other hand, organizations should grant work autonomy to individuals and teams when designing work or forming teams. Job autonomy directly correlated with job crafting, and employees with a growth mindset exceled in job crafting when job autonomy was high. Consequently, organizations should fully empower teams to select their own work methods, procedures, and time. Team leaders should also permit employees greater autonomy in decision-making, enabling them to flexibly arrange their work. To sum up, job autonomy should be emphasized in job design, particularly in roles requiring innovation and flexibility. By granting employees more control over their work methods and schedules, organizations can enhance the expression of growth mindsets, leading to higher levels of job crafting and productivity.

These strategies are particularly relevant in knowledge-intensive industries, where job crafting is critical for innovation and continuous improvement. Managers in such contexts should focus on creating environments that balance autonomy with structured guidance, ensuring employees feel supported while maintaining flexibility.

### 5.3. Limitations and Future Prospects

This study has theoretical and practical implications but also limitations for future research. First, the sample size is adequate but lacks generalizability. While the online platform used was convenient, economic, and diverse, it may suffer from inattention, self-selection bias, and social approval effects [80]. Thus, more field studies are needed in the future. Additionally, our study lacks explicit consideration of age and gender diversity in the interpretation of the results. It is possible that the effects of growth mindset on job crafting differ across different age groups or between genders. Future research should explore these factors in greater detail to determine their moderating role.

Second, this study lacks longitudinal data and objective data. Our study did not fully address the impact of long-term changes (e.g., those related to aging) or spontaneous adaptations to unexpected circumstances. Future research should design longer longitudinal studies or intervention studies to explore how the growth mindset interacts with these dimensions and investigate the long-term impact of a growth mindset on job crafting. Moreover, we did not consider physical or psychological limitations that could hinder some individuals’ ability to engage in job crafting. Chronic health conditions, mental health issues, or cognitive impairments may influence the degree to which an individual can apply a growth mindset to their job. Future research should account for such factors to provide a more comprehensive understanding of the conditions under which a growth mindset influences job crafting.

Third, while this study explored the moderating role of job autonomy in the growth mindset–job crafting relationship, other moderators and mediators might be involved. Future research could delve into moderators like organizational climate and team interdependence. Additionally, employees’ growth mindset could foster proactive motivation or shape meaning systems. Future studies could explore mediating mechanisms through proactive motivation models, self-regulation theory, or other frameworks.

Fourth, the findings of this study may be influenced by cultural context. For example, cultural orientation, such as long-term orientation, power distance, and uncertainty avoidance, could moderate the activation of growth mindset and its influence on job crafting [81]. Future research could explore these cross-cultural differences to assess the generalizability of the findings. This would provide deeper insights into how organizational strategies can be adapted to diverse cultural settings.

## 6. Conclusions

This study demonstrates that a growth mindset positively influences job crafting, particularly when employees have higher levels of job autonomy. Both Study 1 (situational experiment) and Study 2 (longitudinal survey) show that individuals with a growth mindset are more likely to engage in job crafting, especially when they have greater autonomy over their work. These findings extend previous research on the growth mindset and proactive behaviors by emphasizing the moderating role of job autonomy. This study extends TAT, highlighting how job autonomy activates the relationship between a growth mindset and job crafting. Practically, organizations can enhance employee engagement and performance by fostering a growth mindset and providing greater job autonomy. However, this study has limitations, particularly the lack of consideration for how age, gender, and individual physical or psychological limitations may influence job crafting. Future research should address these factors and explore the long-term effects of growth mindset and job autonomy on employees’ positive organizational behaviors, performance, and well-being.

## Figures and Tables

**Table 1:** Manipulation test.

Groups	N	M	SD	t
Groups of growth mindset	83	5.07	0.64	19.39 ***
Groups of fixed mindset	97	2.75	0.91	
Groups of high job autonomy	95	4.48	0.37	32.62 ***
Groups of low job autonomy	85	1.73	0.72	

Note. *** *p <* 0.001.

**Table 2:** Job crafting scores for each experimental group.

Groups	N	M	SD	F	*p*
Fixed mindset × low job autonomy	48	2.47	0.91	0	0.989
Growth mindset × low job autonomy	37	2.47	1.22		
Fixed mindset × high job autonomy	49	4.15	0.73	37.85 ***	0.000
Growth mindset × high job autonomy	46	5.23	0.47		

Note. *** *p <* 0.001.

**Table 3:** Demographic characteristics of the valid sample.

Characteristics	Categories	Frequency	Percentage (%)
Gender	Male	77	32.6
	Female	159	67.4
Age	18–25	21	8.9
	26–35	176	74.6
	36–45	27	11.4
	Above 46	12	5.1
Education	Associate degree and below	21	8.9
	Bachelor’s degree	164	69.5
	Master’s degree and above	51	21.6

**Table 4:** Descriptive statistics and correlation coefficients.

Variables	M	SD	1	2	3	4	5
1 Age	31.87	6.35					
2 Gender	0.67	0.47	−0.21 **				
3 Education	2.13	0.54	−0.26 **	0.18 **			
4 Growth mindset	4.99	0.72	−0.01	0.07	−0.04		
5 Job autonomy	4.21	0.63	−0.03	0.06	0.13 *	0.39 **	
6 Job crafting	4.07	0.48	0.03	0.04	0.03	0.42 **	0.51 **

Note. M = mean; SD = standard deviation; * *p <* 0.05 (two-tailed test), ** *p <* 0.01 (two-tailed test); gender (“0” = male, “1” = female); education (“1” = associate degree and below; “2” = bachelor’s degree, “3” = master’s degree and above).

**Table 5:** Regression analysis of growth mindset and job crafting with moderation effect of job autonomy.

Variables	Model 1		Model 2		Model 3	
	β	SE	β	SE	β	SE
Gender	0.04	0.07	0.00	0.06	−0.01	0.06
Age	0.03	0.05	0.02	0.05	0.00	0.04
Education	0.03	0.06	0.05	0.06	−0.00	0.05
Growth mindset			0.42 ***	0.04	0.22 ***	0.04
Job autonomy					0.37 ***	0.05
Growth mindset × Job autonomy					0.17 ***	0.05
R^2^	0.00		0.18		0.35	
F	0.23		12.25 ***		20.87 ***	

Note. The dependent variable is job crafting. “Job autonomy” represents the main effect of job autonomy, while “Growth mindset × Job autonomy” represents the interaction term. Independent variable and moderator variable were centered for analysis. *** *p <* 0.001.

## Data Availability

The data supporting the results of this study are available at https://osf.io/fzmjd/ (accessed on 23 May 2024).
